# Exploring Calcium Manganese Oxide as a Promising Cathode Material for Calcium-Ion Batteries

**DOI:** 10.1021/acs.chemmater.3c00659

**Published:** 2023-10-06

**Authors:** Paul Alexis Chando, Sihe Chen, Jacob Matthew Shellhamer, Elizabeth Wall, Xinlu Wang, Robson Schuarca, Manuel Smeu, Ian Dean Hosein

**Affiliations:** † Department of Biomedical and Chemical Engineering, Syracuse University, Syracuse, New York 13244, United States; ‡ Department of Physics, Binghamton University State University of New York, Binghamton, New York 13902, United States

## Abstract

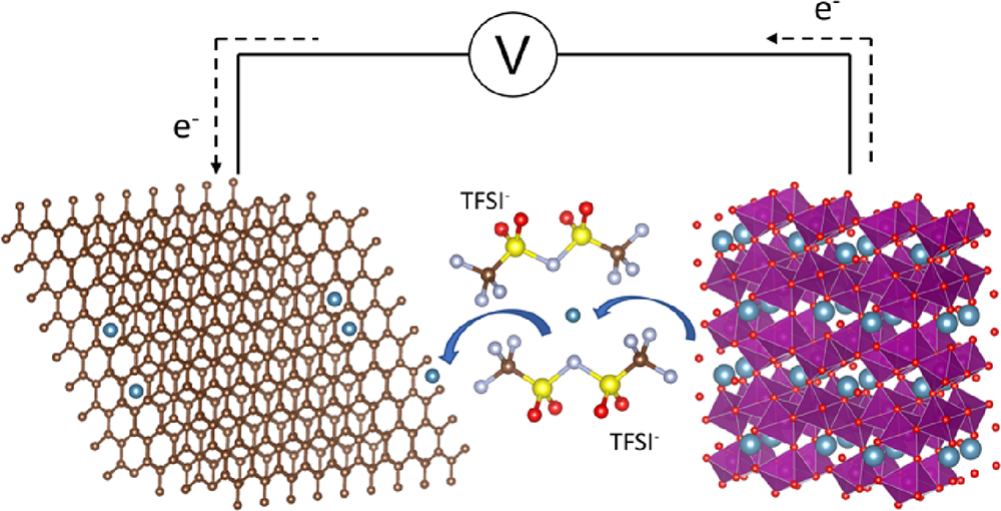

The dependence on lithium for the energy needs of the world, coupled with its scarcity, has prompted the exploration of postlithium alternatives. Calcium-ion batteries are one such possible alternative owing to their high energy density, similar reduction potential, and naturally higher abundance. A critical gap in calcium-ion batteries is the lack of suitable cathodes for intercalating calcium at high voltages and capacities while also maintaining structural stability. Transition metal oxide postspinels have been identified as having crystal structures that can provide low migration barriers, high voltages, and facile transport pathways for calcium ions and thus can serve as cathodes for calcium-ion batteries. However, experimental validation of transition metal oxide postspinel compounds for calcium ion conduction remains unexplored. In this work, calcium manganese oxide (CaMn_2_O_4_) in the postspinel phase is explored as an intercalation cathode for calcium-ion batteries. CaMn_2_O_4_ is first synthesized via solid-state synthesis, and the phase is verified with X-ray diffraction (XRD). The redox activity of the cathode is investigated with cyclic voltammetry (CV) and galvanostatic (GS) cycling, identifying oxidation potentials at 0.2 and 0.5 V and a broad insertion potential at −1.5 V. CaMn_2_O_4_ can cycle at a capacity of 52 mAh/g at a rate of C/33, and calcium cycling is verified with energy-dispersive X-ray spectroscopy (EDS) and X-ray photoelectron spectroscopy (XPS) and modeled with density functional theory (DFT) simulations. The results from the investigation concluded that CaMn_2_O_4_ is a promising cathode for calcium-ion batteries.

## Introduction

The increasing demand for new technologies that rely on lithium-ion batteries, coupled with the limited global supply of lithium, underscores the urgent need to explore alternative battery chemistries that offer a sustainable and economically viable solution to the growing energy demands of the world. Calcium (ion) batteries are a particularly promising alternative due to their high volumetric capacity of 2073 mAh/cm^3^ and low standard reduction potential of −2.87 (vs SHE). Additionally, calcium is the fifth most abundant element in Earth’s crust, with abundant domestic supply in the US and China (two major battery manufacturers), making the economics associated with its raw materials relatively inexpensive.^1−3^ Compared to magnesium, calcium has a larger ionic radius, causing calcium to have a lower charge density that is more amenable to intercalation chemistry.^4^ Calcium-ion batteries would be suitable for the same technologies that employ Li-ion technology, including portable electronics, stationary storage, and even electric vehicles, owing to their combination of high capacity, high voltage, and low cost.

The development of calcium-based batteries has a set of challenges that must be addressed. One prominent issue is the identification of suitable cathodes. Cathodes for calcium batteries must possess certain properties including high capacity, ability to run at high voltage, structural stability during cycling, and possessing stable transport pathways for calcium diffusion in and out of the structure.^1,5^ To facilitate the search for such candidates, density functional theory (DFT) calculations have been used as a screening tool, incorporating the previously described criteria in its search. Results from DFT screening have identified transition metal oxides as promising candidates for calcium batteries.^6−8^ So far, however, only a select few of these compounds have been experimentally investigated.^9−12^ Those that have been evaluated have shown promising electrochemical activity with reversible insertion and deinsertion of calcium. These results have prompted an investigation into similar compounds.

One transition metal oxide that has not been thoroughly studied is CaMn_2_O_4_. Early DFT investigations concluded that the spinel structure of CaMn_2_O_4_, with a cubic crystal structure, would be an attractive option as a cathode due to its low migration barrier of 600 meV since calcium would be tetrahedrally coordinated.^13,14^ However, true spinels of CaMn_2_O_4_ are difficult to synthesize, requiring high pressures to produce a true spinel structure.^15^ The more naturally occurring alternative is the postspinel of CaMn_2_O_4_. Postspinels are crystal structures derived from spinels after being subjected to high temperature and pressure, causing the cubic crystal system to change and typically densify. DFT calculations performed on the postspinel structure have concluded that while manganese itself is a transition metal capable of accessing different oxidation states, the diffusion barrier of 1.8 eV associated with calcium’s migration between octahedral sites is problematic.^16,17^ Preliminary experimental investigations of the postspinel using an electrolyte of Ca(BF_4_)_2_ in EC/PC at 75 °C did not yield any structural changes via XRD and no other experimental work has been performed on the postspinel CaMn_2_O_4_.^17^

Herein, we report an experimental evaluation of the postspinel CaMn_2_O_4_ for calcium-ion batteries. The calcium manganese oxide is prepared using a solid-state synthesis method and its phase is verified through Rietveld refinement of XRD.^15,18^ The synthesized CaMn_2_O_4_ is processed into an electrode where its electrochemical activity is evaluated in an electrolyte of Ca(TFSI)_2_ in DME using cyclic voltammetry, linear sweep voltammetry, and galvanostatic cycling. The redox activity of manganese and cycling of calcium are analyzed using XRD, XPS, and scanning electron microscopy (SEM), complemented with DFT calculations. The results from these experiments and simulations outline that the postspinel can successfully cycle calcium at room temperature.

## Experimental Section

### Materials

Manganese oxide (MnO_2_), calcium carbonate (CaCO_3_), diethylene glycol monobutyl ether (DME), ferrocene, and 1-methyl-2-pyrrolidone (NMP) were purchased from Sigma-Aldrich, USA, and used as received. Calcium(II) bis(trifluoromethanesulfonyl)imide (Ca(TFSI)_2_) was purchased from Solvionic. Silver nitrate (AgNO_3_) was purchased from Alfa Aesar. Ca(TFSI)_2_ salt was vacuum-dried at 120 °C overnight before use. Polytetrafluoroethylene (PTFE) preparation (60 wt %) was purchased from Sigma-Aldrich. Polyvinylidene fluoride (PVDF) binder, super P carbon black, activated carbon, CR2032 316 stainless steel coin cells, and aluminum foil (∼10 μm in thickness) were purchased from MTI Corporation, USA.

### Synthesis of CaMn_2_O_4_

A solid-state synthesis method was used for postspinel CaMn_2_O_4_. MnO_2_ and CaCO_3_ were combined in a 7:3 Mn:Ca molar ratio to produce a total mixture of 20 g. The powders were mixed in a ball mill for 1 h at 200 rpm. The mixture was then baked in a furnace for 20 h at 1100 °C. The calcined product was ground with a mortar and pestle and ball-milled under the previous conditions. After ball milling, the powder was baked for 20 h at 1200 °C. Following the bake, the same grinding and ball milling process was repeated. The last three bakes were performed at 1250 °C for 20 h. After each bake, the grinding and ball milling procedure was followed.^15,18^

### Electrode Preparation

The electrodes used in the study were formulated by using two methods. Electrodes used with the in situ cell and cyclic voltammetry experiments were processed as freestanding electrodes, while galvanostatic experiments using coin cells were assembled with slurry casted electrodes. To prepare a freestanding electrode, powders of CaMn_2_O_4_ (65 wt %) and carbon black (5 wt %) were mixed with PTFE binder (30 wt %).^19,20^ The active material was mixed with carbon black in a ball miller. The components were mixed at 19.67 Hz for 90 min.^21^ After ball milling, the mixture was placed in a mortar and pestle, where PTFE binder was added and suspended in excess ethanol. The materials were mixed until all excess ethanol evaporated. The freestanding electrode would then be processed in a hot roll press at 80 °C. Freestanding electrodes were then vacuum-dried overnight at 80 °C for a final weight density of 3 mg/cm^2^. Activated carbon freestanding electrodes were prepared by using the same procedure. The composition of the activated carbon electrodes was activated carbon (80 wt %) and PTFE binder (20 wt %) with a mass loading density of 30 mg/cm^2^.^22^

The following procedure was used for the processing of casted electrodes in galvanostatic experiments. To prepare the working electrode, a slurry of 95% CaMn_2_O_4_, 2.5% carbon black, and 2.5% PVDF binder was made. The solid content of the slurry is fixed at 65 wt %.^23,24^ The active material and conductive additive were mixed in powder form for 60 min in the ball mill at 19.67 Hz. Separately, PVDF and 2/5 of the total NMP were mixed in the ball mill for 30 min and then sonicated for another 30 min. The premixed solids were added and mixed again in the ball mill and sonicator. One-third of the remaining NMP, or 1/5 of the overall volume, was added and the ball mill and sonication mixings were repeated. The remaining NMP was added in 1/5 increments with the ball milling and sonication mixings following each addition.^25^ Once mixed, the slurry was cast onto the carbon-coated aluminum current collector with a thickness of 200 μm. After the slurry was casted onto the current collector, the casted electrode was dried at 80 °C for 1 h and subsequently vacuum-dried at 110 °C overnight.^26^ The method for making the cast activated carbon was the same as CaMn_2_O_4_ with the exception that the activated carbon was cast onto a carbon-coated copper collector. Mass loading of the CaMn_2_O_4_ working electrode was recorded to be 5 mg/cm^2^, while the activated carbon mass loading was recorded to be 30 mg/cm^2^.

### Electrolyte Preparation

The electrolyte used in all electrochemistry experiments was 0.5 M Ca(TFSI)_2_ in DME. A stock of the DME solvent was prepared by drying it for 48 h over 3 Å molecular sieves. To remove any residual moisture from the salt, the Ca(TFSI)_2_ salt was dried overnight at 120 °C in a vacuum oven. Following the drying, the salt was then dissolved in DME and magnetically stirred for 24 h to allow homogenization of the salt in DME. The electrolyte was then dried over 3 Å molecular sieves again to remove any remaining traces of water, and the water content in the electrolyte was verified to be below 50 ppm using a Karl Fischer Titrator (899 Coulometer, Metrohm). Experiments involving the calibration of the Ag/Ag^+^ reference electrode included the addition of dried 50 mM ferrocene to the Ca(TFSI)_2_ DME electrolyte along with the addition of 0.01 M AgNO_3_ supporting salt in the reference electrode.^27^ The use of two electrolytes for the reference electrode was designed to avoid the development of junction potentials between the reference electrode and the bulk electrolyte.

### Electrochemistry

Cyclic voltammetry (CV) and linear sweep voltammetry (LSV) experiments were performed in beaker cell setups (Gamry Instruments). Cyclic voltammetry experiments used a three-electrode configuration with Ag/Ag^+^ reference electrode (Redox.me), CaMn_2_O_4_ working electrode, and activated carbon as the counter electrode. Working and counter electrodes were attached to the end of 316 stainless steel supports and immersed in electrolyte. The experiments were carried out for 20 cycles at a scan rate of 0.5 mV/s and performed with a Metrohm Autolab. Calibrations of the Ag/Ag^+^ reference electrode and activated carbon with ferrocene were also carried out in a beaker cell with platinum wire as the working electrode (Figure S1a,b). The Ag/Ag^+^ reference electrode consists of 0.5 M Ca(TFSI)_2_ in DME along with 0.01 M AgNO_3_. Linear stability window measurements used a two-electrode cell with stainless steel as the blocking electrode and calcium as the nonblocking electrode. Linear scans were made at a sweep rate of 0.5 mV/s from 0 to 2.5 V (Figure S2). All experiments were carried out in an argon-filled glovebox with H_2_O and O_2_ levels maintained below 0.5 ppm.

Galvanostatic and in situ EIS experiments were carried out in two-electrode coin cell configurations with CaMn_2_O_4_ as the working electrode (5 mm diameter) and activated carbon as the counter electrode (15 mm diameter). Eighty microliter portion of Ca(TFSI)_2_ in DME was used in each coin cell assembly, and all coin cells were assembled in a glovebox. Testing of all coin cells began with a 2 h open circuit voltage to allow components to equilibrate before testing. Galvanostatic cycling of coin cells was performed on an Arbin battery tester where the coin cells were cycled at room temperature at a rate of C/33. In situ EIS measurements were performed with a Solartron Energy Lab XM Instrument over a frequency range from 0.1 Hz to 1 MHz between each charge and discharge of the coin cell. A 10 mV perturbation was used in all EIS measurements and the resulting Nyquist plot was analyzed using an equivalent circuit model.^28−31^

### Electrode Characterization

A Rigaku Miniflex diffractometer was used for the collection of the X-ray diffraction data. Copper (Cu) Kα radiation was used to collect data in the range of 10–80° at a scan rate of 5°/min. To eliminate the effect of residual electrolyte, all samples were rinsed with solvent and dried before measurements were taken. The XRD data collection was performed at room temperature. In situ XRD measurements were performed with a custom in situ cell (Figures S3 and S4). Figure S3a is a schematic of the in situ cell, and Figure S3b is the assembled in situ cell. Figure S4a outlines the assembled in situ cell as it is installed inside of the Rigaku Miniflex diffractometer, while Figure S4b is a baseline XRD scan of CaMn_2_O_4_ within the in situ cell. Rietveld refinement of each material was performed using Highscore Plus software.^32^ The Materials Project was used for the identification of phases.^33^ Scanning electron microscopy (SEM) was performed using a JEOL 5600 instrument and was equipped with an energy-dispersive X-ray (EDX) detector. The accelerating voltage used on all samples was 15 kV. All samples were first sputter-coated with gold before being placed inside the SEM chamber. XPS analysis of the CaMn_2_O_4_ electrodes was performed at the Cornell Center for Materials Research (CCMR) with a Thermo Nexsa G2 XPS Surface Analysis System that employs monochromatic Al Kα X-ray operating at 10 mA and 12 kV. Samples were mounted using conductive spring clips in contact with the top surfaces of the samples. High-resolution scans of the Mn 2p, Mn 3s, O 1s, and C 1s lines were performed with an analysis spot of 400 μm in diameter, a step size of 0.1 eV, and a pass energy of 50 eV. Survey scans were performed with a pass energy of 200 eV and a step size of 0.4 eV.

### Ab Initio Calculations

The DFT calculation was performed using the Vienna Ab Initio Simulation Package,^34^ which uses the projector augmented wave method.^35^ The PBE + *U*(36) functional was chosen for electronic interactions with a *U* value of 3.9 eV. The cutoff energy for the plane-wave basis is 700 eV, and the *k*-point grid is a 15 × 5 × 5 mesh with a Γ-centered scheme. *k*-Point and cutoff energy were tested, and results converge to 1 meV per atom difference. Antiferromagnetic configurations were chosen for all cases. This configuration is kept for all other calculations. Each structure was fully relaxed, including energies, cell parameters, and atomic positions. All deintercalation of Ca ions was performed using the fully relaxed CaMn_2_O_4_ structure and was then relaxed again to obtain correct energies, cell parameters, and atomic positions.

## Results and Discussion

**Figure 1:**
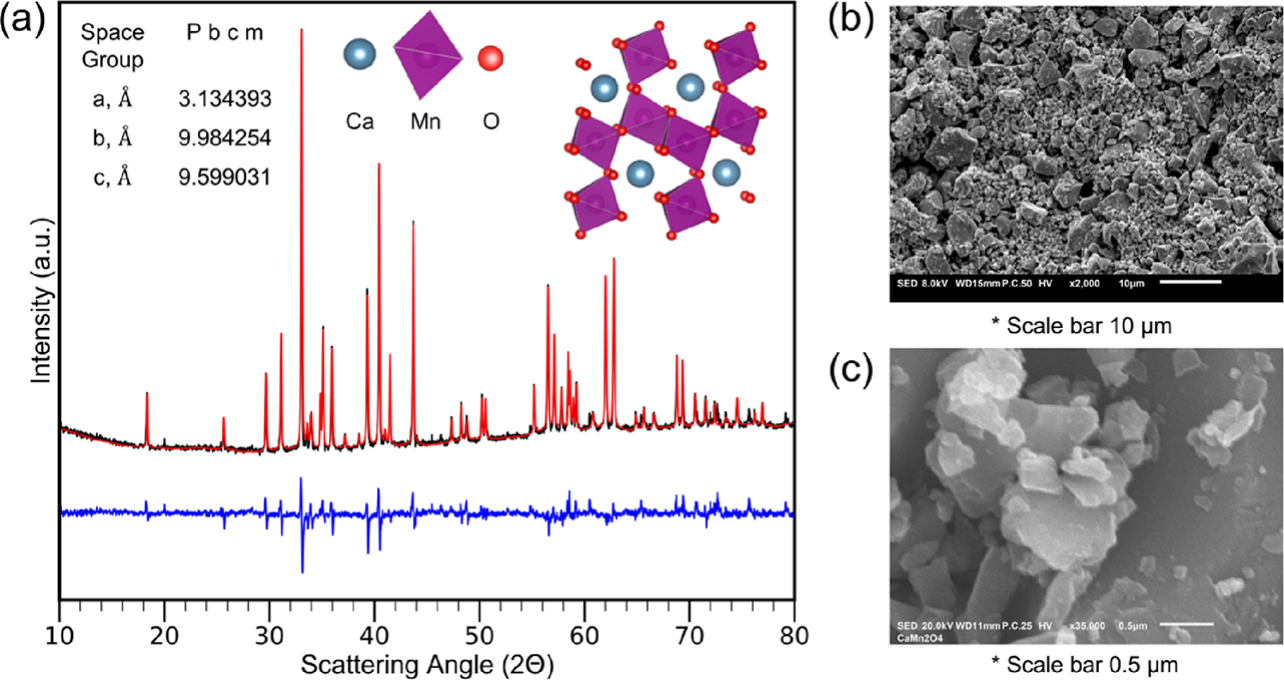
Synthesis of postspinel CaMn_2_O_4_. (a) XRD diffraction of CaMn_2_O_4_ (inset of lattice parameters, space group and unit cell). (b) SEM image of CaMn_2_O_4_ powders (scale bar, 10 μm). (c) Close-up SEM image of CaMn_2_O_4_ particles (scale bar, 0.5 μm).

The synthesized calcium manganese oxide was identified with XRD. Figure 1a displays the XRD profiles of the experimental and fitted results from Rietveld refinement performed on the synthesized CaMn_2_O_4_ powder. The refinement on the sample concluded a pure postspinel phase with high crystallinity and no remaining competing phases from the solid-state synthesis. The results from the refinement identify an orthorhombic structure with a space group of *Pbcm* (mp-18844).^37^ The resulting lattice parameters were *a* = 3.1575 Å, *b* = 9.9916 Å, and *c* = 9.6738 Å. The Scherrer equation performed on the synthesized powder found a crystallite size that was approximately 600 Å.^38^ SEM analysis of the synthesized powder (Figure 1b,c) found the morphology of the particles to have a polydisperse, irregular morphology.^39^

**Figure 2:**
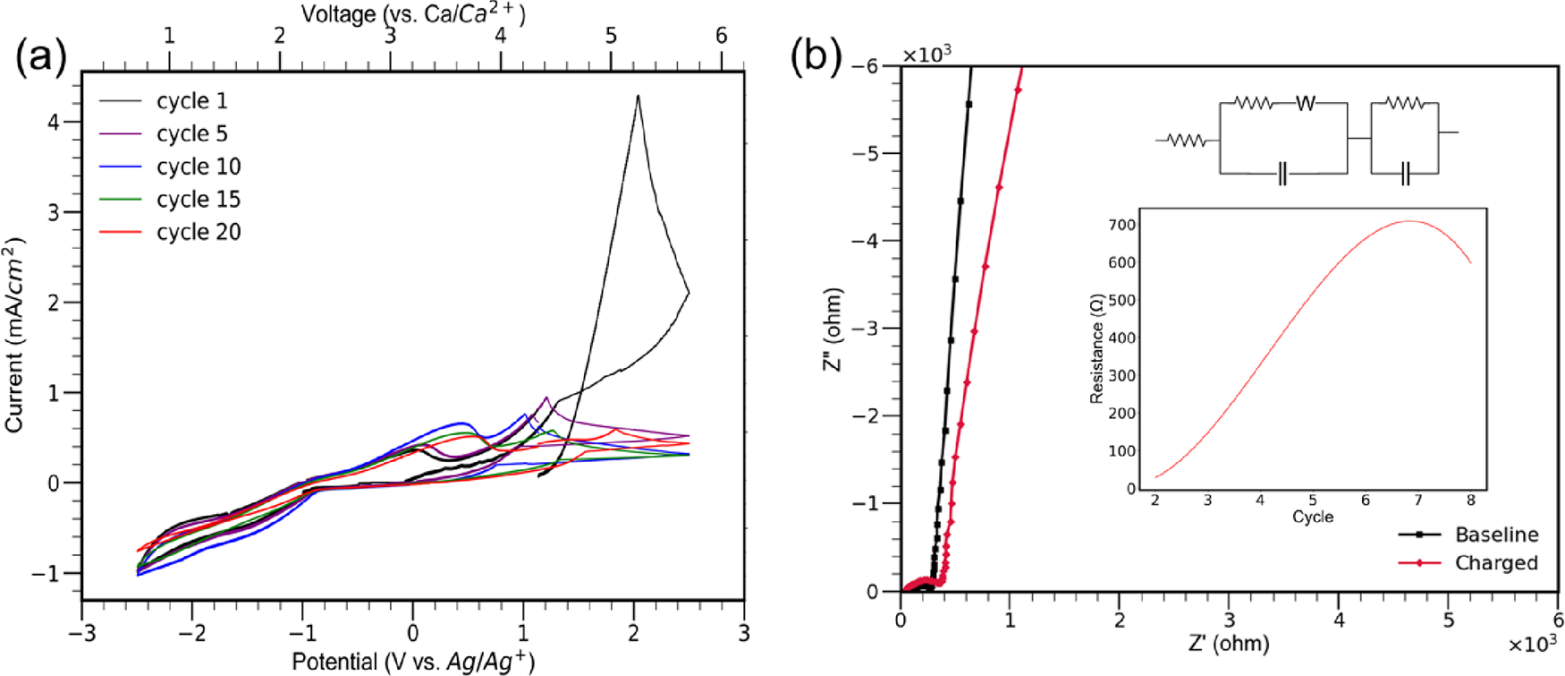
(a) Cyclic voltammetry of CaMn_2_O_4_. (b) In situ EIS measurements of CaMn_2_O_4_ (inset shows an equivalent circuit used for impedance analysis and charge transfer resistance plot).

Evaluation of the redox activity for CaMn_2_O_4_ began with cyclic voltammetry. A three-electrode cell was used for the analysis. The freestanding electrodes of CaMn_2_O_4_ and activated carbon were assembled in a beaker cell with Ag/Ag^+^ being used as a reference electrode. Calibrations of both the Ag/Ag^+^ electrode and activated carbon are summarized in Figure S1. Open circuit voltage (OCV) measurements of the cell were made before electrochemical testing and found the cell to have an OCV of 0.275 V vs Ag/Ag^+^. The results from cyclic voltammetry outline redox active processes with CaMn_2_O_4_ where an oxidative potential can be observed at 0.2 V (Figure 2a). Upon continued cycling, the oxidative potential shifts from 0.2 V to approximately 0.5 V, indicative of an irreversible process or partial phase change from CaMn_2_O_4_ undergoing a conversion reaction.^40,16^ The oxidation potential does stabilize at 0.5 V by the end of cycling and is consistent with previously reported oxidation potentials of manganese.^31^ There is also evidence of limited reinsertion of calcium back into the CaMn_2_O_4_ electrode. There is a broad reduction peak observed at −1.5 V.^31,41^ The lack of a clear cathodic peak indicates that the reinsertion of calcium into the cathode is slow.^42^ With continued cycling of the electrode, the reduction peak at −1.5 V diminishes until the feature is absent by the 20th cycle and only a negative sloping line exists in the cathodic trace of the CV. This behavior outlines the limited success in reducing manganese from its +4 to +3 oxidation state with the intercalation of calcium.^43^ The results from the CV also produce a secondary peak that drifts between 1.5 and 2 V and is observed on the first cycle at 2 V. However, this peak is not related to the redox activity of manganese and is instead a product of the electrolyte interaction with the 316 stainless steel electrode supports. A linear stability window of the electrolyte was performed and found behaviors consistent with this peak in the cyclic voltammetry experiment (Figure S2).

One of the major issues known to the postspinel structure of CaMn_2_O_4_ is the charge transfer resistance that develops from calcium attempting to diffuse through the crystal structure of the electrode. Previous DFT investigations into the postspinel CaMn_2_O_4_ have revealed that a substantial diffusion barrier exists for the electrode that suppresses large capacities of calcium from being cycled in and out of the electrode.^16,17^ To establish the degree of the charge transfer resistance experienced by CaMn_2_O_4_, in situ EIS studies were performed on the electrode in coin cell configurations.^28−31^ The Nyquist plots generated from the in situ EIS experiments can be seen in Figure 2b and Figure S5. The impedance data was fit to an equivalent circuit, which can be seen in an inset of Figure 2b. The resulting resistances from cycling CaMn_2_O_4_ are an inset as well with Figure 2b. The charge transfer resistance increases substantially over the course of cycling, reaching a maximum resistance of 700 ohm. The increase in resistance is consistent with the previously reported sluggish diffusion kinetics of the postspinel structure and has been observed with a decreased charge capacity with initial cycles.^31^ By cycle 8 though, a drop in the resistance can be seen, with the value decreasing from 700 to 600 ohm. This decrease in resistance allows for better intercalation chemistry in CaMn_2_O_4_. Upon continued cycling, the charge transfer resistance continued to decrease, and comparisons of cycling data can be seen in Figure S5. This decrease in charge transfer resistance in CaMn_2_O_4_ is suspected to be partly influenced by some irreversible process or change in CaMn_2_O_4_ that has already been identified with the CV experiment.

**Figure 3:**
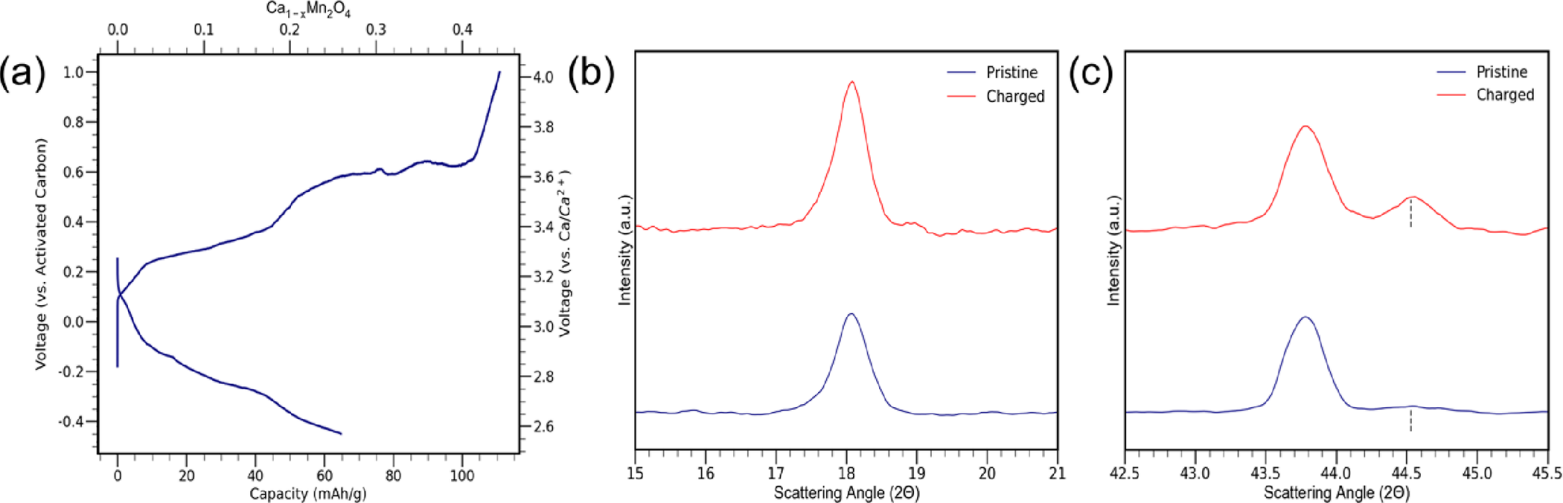
(a) Maximum charge of CaMn_2_O_4_ (C/200) and discharge. (b) XRD of lattice plane (020). (c) XRD of lattice plane (132) before and after charging (dashed lines denoting emergence of peak at 44.5°).

To determine the maximum capacity of calcium that could be extracted from calcium manganese oxide, a maximum charge was performed on it. Freestanding electrodes of CaMn_2_O_4_ and activated carbon were used in the analysis and charged at a rate of C/200. The results from the maximum charge revealed that a total of 100 mAh/g could be extracted from CaMn_2_O_4_ (Figure 3a). Similar experiments aimed at performing maximum charges on a spinel structure have yielded the same two-tiered charging profile that has been labeled as some partial phase transformation.^44^ There are small fluctuations in the voltage of the second plateau at approximately 0.6 V. There are several possible causes for this, including nonuniform electrolyte distribution and variations in electrode roughness, causing nonuniform charging. The sharp rise in voltage after 100 mAh/g to 4 V indicates that the capacity of the cathode has been exhausted. Based on DFT calculations and the literature with magnesium-based spinel structures, the phase change observed in the charging profile would be a result from conversion reactions that form decalciated CaMn_2_O_4_ and Mn_3_O_4_ phases. The DFT calculations performed on CaMn_2_O_4_ are summarized in Figures S6–S8 and Tables S1–S8. Figure S6a outlines the voltage profile of CaMn_2_O_4_, while Figure S6b is the convex hull of CaMn_2_O_4_ as it undergoes intercalation chemistry. Based on the established convex hull of CaMn_2_O_4_, most of the formation energies lie slightly above the hull, identifying a preference for the postspinel cathode to undergo conversion reactions.^16^ Based on the magnesium battery literature, the lower voltages from the charging of CaMn_2_O_4_ would be identified as intercalation behavior involving the extraction of calcium from the postspinel, while the second plateau would focus more on conversion reactions of the postspinel cathode.^16,17,42,45−52^ XRD analysis was performed on the working electrode before and after charging. The refinement performed on CaMn_2_O_4_ tracked lattice parameter changes of CaMn_2_O_4_ and occupancies of calcium. The pristine sample of CaMn_2_O_4_ was refined with lattice parameters *a* = 3.156676 Å, *b* = 9.988452 Å, and *c* = 9.675391 Å. The charged sample was analyzed to have lattice parameters *a* = 3.154765 Å, *b* = 9.978689 Å, and *c* = 9.666233 Å. The occupancies of calcium were also tracked before and after charging. The occupancy of calcium in CaMn_2_O_4_ post charging decreased from 1.0 to 0.785. In addition to the decalciated CaMn_2_O_4_ phase, Mn_3_O_4_ (mp-18759) and MnO (mp-19006) phases were also identified and refined.^53,54^ The Mn_3_O_4_ phase was refined with lattice parameters *a* = 5.761503 Å, *b* = 5.761503 Å, and *c* = 9.464502 Å, while the MnO phase was refined with lattice parameters *a* = *b* = *c* = 4.48 Å. The Mn_3_O_4_ phase was the product of a conversion reaction, while the MnO phase occurred from a disproportionation reaction occurring on the surface of the CaMn_2_O_4_ electrode.^16,17,45,55,56^ Results from the Rietveld refinement performed on the CaMn_2_O_4_ cathode post charging are summarized in Figure S9a and Tables S9–S11. Previous DFT calculations performed on the CaMn_2_O_4_ postspinel have reported that successful intercalation chemistry with the structure would require volume variations of CaMn_2_O_4_ to be less than 6%.^17,57^ DFT calculations of CaMn_2_O_4_ yielded a volumetric reduction of 3% for the removal of 25% of calcium. Comparisons of the experimental lattice parameters produce a volume variation of approximately 0.25%. The small volumetric variations of CaMn_2_O_4_ post charging are a feature that has been observed with MgMn_2_O_4_ spinels when two phases were present in the cathode as a result of conversion reactions.^46−48^ In addition to performing Rietveld refinement of the charged CaMn_2_O_4_ cathode, several XRD changes in the electrode were observed that aligned with DFT calculations. The scope of structural changes of the cathode and their comparisons to theoretical XRD patterns, where 25% of calcium was removed, are summarized in Figure S10. Figure S10a compares the experimental XRD patterns between the pristine and charged samples, while Figure S10b compares theoretical XRD patterns generated from DFT calculations. Theoretical XRD patterns of decalciated CaMn_2_O_4_ are also outlined in Figure S8. One of the most prominent changes with CaMn_2_O_4_ was an increase in the intensity of the (020) reflection (Figure 3b). The (020) lattice plane intensity increase is a feature consistent with DFT calculations and is associated with activity occurring along the MnO_6_ edge sharing octahedra.^10,40^ The intensity increase of the (020) lattice planes was attributed to the activity of calcium extraction and not a preferred orientation effect since similar effects were not observed with parallel planes.^58^ Another structural development predicted from DFT calculations is the development of two peaks with a (132) reflection (Figure 3c). The emergence of a second peak for the (132) reflection is identified with dashed lines at ∼44.5°, and its intensity increases by the end of charging. The development of two peaks in CaMn_2_O_4_ post charging is likely caused by some decrease in symmetry in the host structure or could be attributed to the formation of a secondary phase in CaMn_2_O_4_ as it undergoes a conversion reaction, forming Mn_3_O_4_.^16,17,48,50,59,60^ Evidence of partial phase changes is further supported by the activity of the (055) reflection. The pristine CaMn_2_O_4_ (055) reflection (Figure S10) has twin peaks, and upon charging, the reflections are consolidated where the second peak is observed as a shoulder rather than an individual peak, consistent with a phase change in the host structure. The structural changes previously outlined between pristine and charged samples of CaMn_2_O_4_ are outlined in Figure S10b with asterisks. Additionally, cyclic voltammetry of CaMn_2_O_4_ (Figure 2a) did produce a shift in the oxidation potential from 0.2 to 0.5 V, consistent with the activity seen in the maximum charge of CaMn_2_O_4_, and would align with some irreversible process with CaMn_2_O_4_. Based on the XRD from the maximum charge, probable developments within CaMn_2_O_4_ would be several domains of the postspinel undergoing conversion reactions, while others transition their symmetries due to Jahn–Teller distortions. Further testing of the electrode with TEM would be needed to confirm crystal structure changes within CaMn_2_O_4_.

In addition to the maximum charge performed on the CaMn_2_O_4_ postspinel, a discharge of 65 mAh/g (Figure 3a) was also performed and subsequently characterized with XRD and Rietveld refinement. A discharge plateau with CaMn_2_O_4_ is observed between 2.9 and 2.75 V (vs Ca/Ca^2+^). The discharge plateau is consistent with the reduction of manganese from +4 to +3 oxidation states based on the magnesium battery literature.^44,46,48^ Following the discharge of CaMn_2_O_4_, the cathode was characterized with XRD and analyzed with Rietveld refinement. The CaMn_2_O_4_ phase was refined with lattice parameters *a* = 3.158088 Å, *b* = 9.997047 Å, and *c* = 9.678633 Å. Occupancy of calcium within the CaMn_2_O_4_ phase was also refined to a value of 0.864617, an increase from the 0.785 occupancy with the maximum charge and identifying a reinsertion of calcium into the postspinel. The Mn_3_O_4_ phase was also identified and refined with lattice parameters *a* = 5.753304 Å, *b* = 5.753304 Å, and *c* = 9.481883. The MnO phase was refined with lattice parameters *a* = *b* = *c* = 4.46448 Å. The results from the analysis performed on the discharged sample are summarized in Table S9b and Tables S9–S11. The Rietveld refinement performed on the cathode supports some intercalation chemistry occurring within the cathode as the calcium occupancy within the phase increases from 0.785 to 0.864617 at the end of discharge.

**Figure 4:**
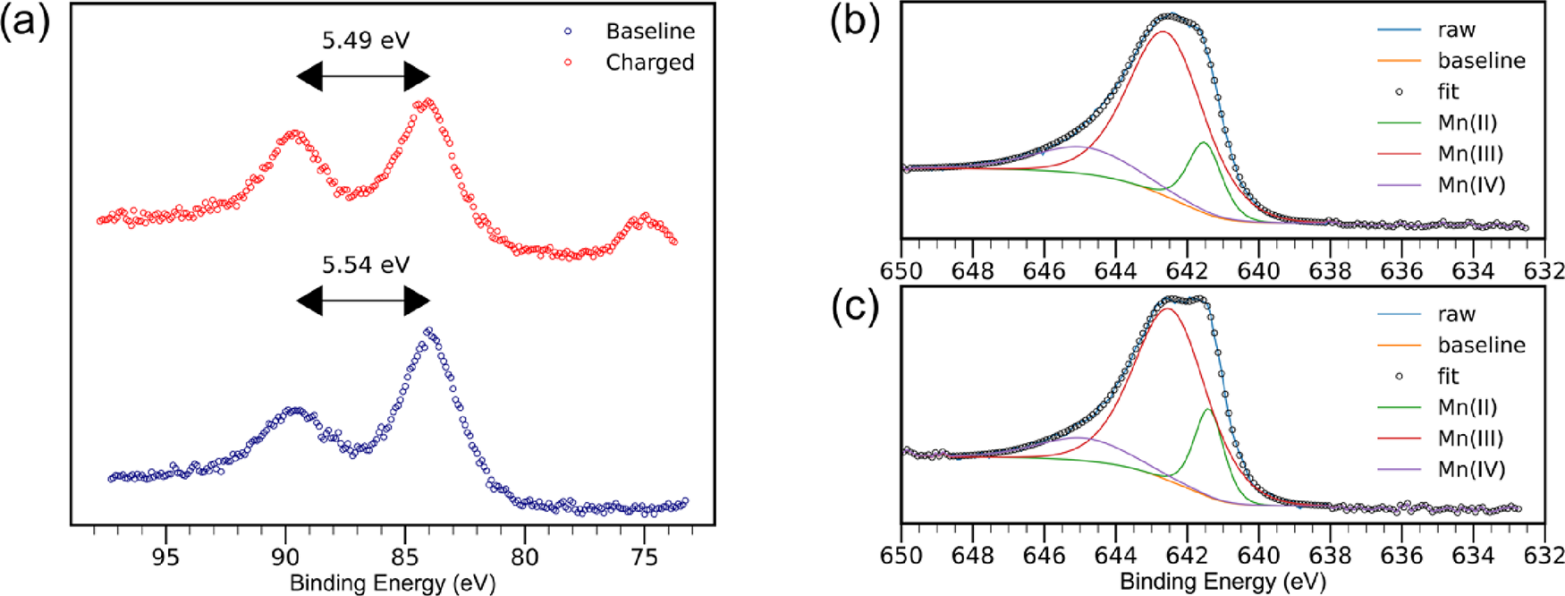
XPS of CaMn_2_O_4_ for average oxidation of manganese: (a) Mn 3s binding energy, (b) Mn 2p binding energy of the charged sample, and (c) Mn 2p binding energy of the baseline sample.

To further corroborate the redox activity associated with the decalciation of CaMn_2_O_4_, we performed XPS analysis to track the average oxidation state change of manganese between pristine and charged samples. To determine the average oxidation state of manganese, high-resolution scans of CaMn_2_O_4_ were performed on Mn 3s, Mn 2p, and C 1s orbitals. The spectra were fitted after applying Shirley background subtractions using nonlinear least squares (implemented in CasaXPS). The charging of CaMn_2_O_4_ was fixed with a C 1s binding energy of 285 eV. Analysis of the Mn 3s orbitals (Figure 4a) gave binding energy differences of 5.54 and 5.49 eV for baseline and charged samples of CaMn_2_O_4_, respectively. Because the average oxidation state of the manganese is established to vary linearly with the binding energies of the 3s orbitals,^61,62^ the average oxidation state of the baseline manganese was found to be 2.94 and the charged sample was 3.01.^63,64^ The average oxidation state of the baseline manganese would be lower than +3 from either oxygen vacancies within the cathode or manganese occupying interstitial sites.^65,66^ To verify the accuracy of the average oxidation states of each sample, the Mn 2p orbitals were analyzed. Deconvolutions of the binding energies were made using a Voigt function with Gaussian–Lorentzian (G/L) contributions. Using established values for the Mn 2p orbital binding energies (Figure 4b,c), the ratios between the manganese oxidation states were found to be in close agreement with the measurement that was made using the Mn 3s orbitals.^61,67^

**Figure 5:**
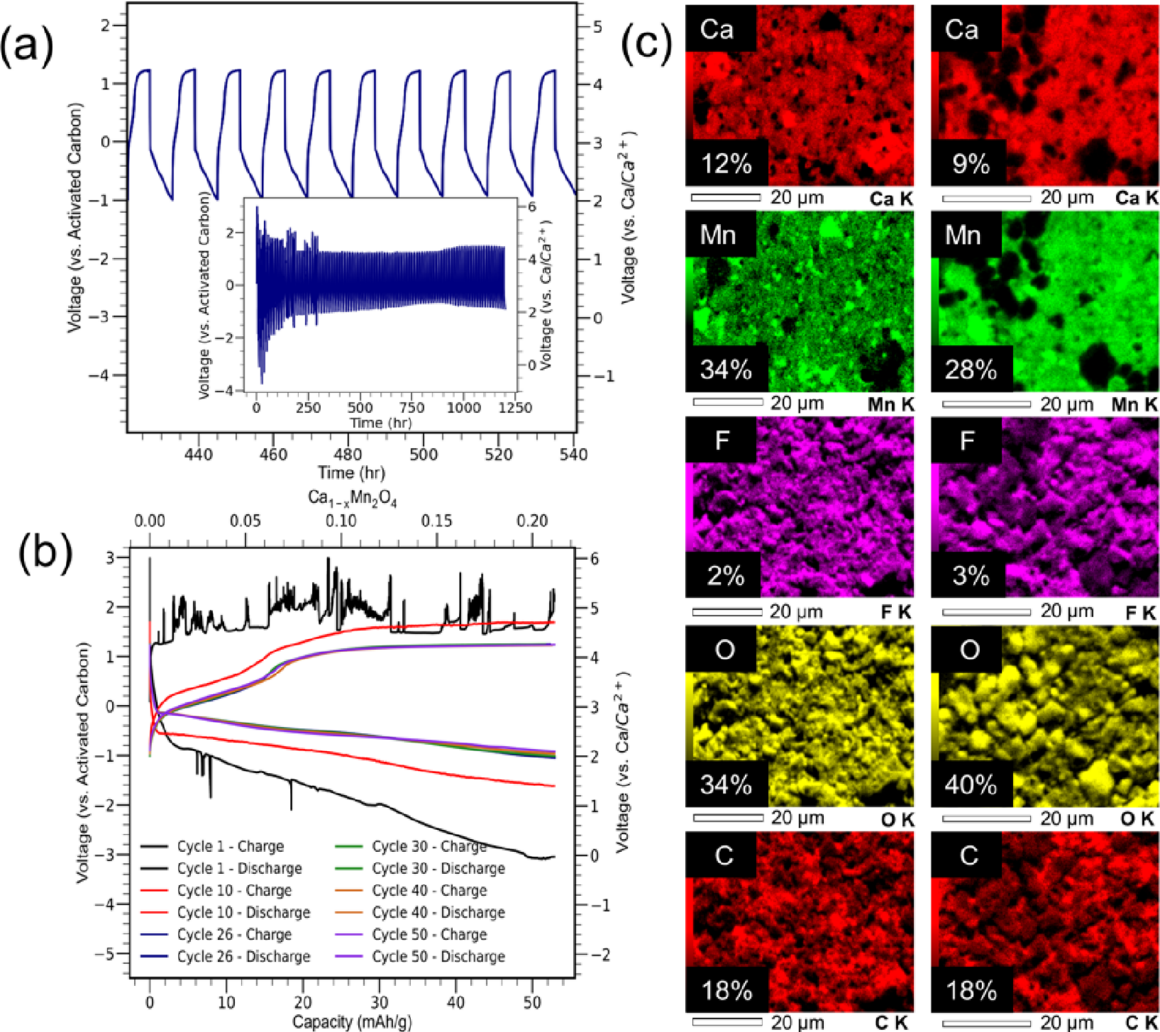
Galvanostatic cycling of CaMn_2_O_4_. (a) Voltage–time curve of selected cycles (inset of full 100 cycle testing with CaMn_2_O_4_). (b) Capacity–voltage curves of selected cycles of CaMn_2_O_4_. (c) EDS of CaMn_2_O_4_ before and after galvanostatic cycling.

After the redox activity of CaMn_2_O_4_ was established, the cathode was then assembled into a coin cell for evaluating long-term cycling behavior at a rate of C/33 for a capacity of 52 mAh/g. The results from the coin cell cycling are outlined in Figure 5a. Analysis of the coin cell cycling with a capacity voltage curve is outlined in Figure 5b. The oxidation of CaMn_2_O_4_ occurs in the voltage region from 0 to 0.5 V and includes a shoulder of redox activity at approximately 0.5 V (∼3.5 V vs Ca/Ca^2+^), consistent with expectations made with cyclic voltammetry studies. The capacity associated with this region of activity is approximately 15 mAh/g. The oxidation of CaMn_2_O_4_ at 3.5 V (vs Ca/Ca^2+^) is a promising first step toward this postspinel system functioning as a high voltage cathode. The termination of CaMn_2_O_4_ oxidation is identified by an additional increase in voltage from ∼0.5 to 1.8 V (∼4.7 V vs Ca/Ca^2+^). There is a plateau approximately at 1.8 V, and a large amount of the coin cell capacity occurs at this voltage. However, this electrochemical activity is not associated with intercalation chemistry of the CaMn_2_O_4_ cathode and is instead a product of electrolyte oxidation.^40^ The first cycle of galvanostatic data does exhibit voltage variations from several factors, including temperature fluctuations within the coin cell and the C/33 charging rate. Other researchers have cycled cathodes at more modest rates below C/50.^17,41^ The higher cycling rate, along with sluggish diffusion kinetics, contributes to voltage spikes in the galvanostatic data. Additional galvanostatic studies using lower cycling rates were performed and exhibited a decrease in the voltage noise as the cathode was cycled. The results of lower cycling rates are outlined in Figure S11.

The first several discharges of CaMn_2_O_4_ include some electrochemical activity observed at −1.5 V, corresponding to the reduction of manganese, and can be observed in cycle 10. Beyond the first 10 cycles of testing, the reinsertion of the calcium back into CaMn_2_O_4_ follows a voltage curve that consistently trends downward. This behavior outlines the reinsertion of calcium back into the electrode but does not include reduction of manganese back into its lower oxidation state.^31,41^

Further confirmation of the calcium activity in the cathode is supported by postmortem EDS and XRD analysis run on CaMn_2_O_4_ at the conclusion of testing. The EDS analysis revealed that the end of cycling produced a decrease in the calcium signal from 12 to 9%. The decrease in the manganese signal can be attributed to the decrease in crystallite size that occurs in the cathode as a function of cycling. Decreased crystallite sizes can translate to a decrease in fluorescence of elements and, as such, cause the elemental signal of manganese to decrease.^68^ Results from the EDS analysis are summarized in Figure 5c. Postcycling XRD of CaMn_2_O_4_ verified the activity of CaMn_2_O_4_ with a decrease in the reflection at 34° (Figure S12). The decrease of this reflection is consistent with DFT calculations performed on CaMn_2_O_4_. The 34° angle of CaMn_2_O_4_ is identified with the (120) lattice plane of CaMn_2_O_4_. Calcium, with a Wyckoff factor of 4d, strongly contributes to the scattering intensity of the (120) lattice plane. The removal of calcium from CaMn_2_O_4_ can be observed with a decrease in the intensity of the (120) lattice plane. XRD analysis of the electrode, using the Scherrer equation, before and after cycling also concluded a decrease in the crystallite size of CaMn_2_O_4_ from 600 to 480 Å. Decreases in crystallite size are correlated to more favorable cycling behavior and its effect can be observed with the evolution of the voltage–capacity curves in Figure 5b.^69^ The energy density from the CaMn_2_O_4_ coin cell cycling behavior was calculated to be 205 Wh/kg, and it falls within the range of energy densities for nickel, manganese, and cobalt (NMC) cathodes for lithium-ion batteries.^70−72^

**Figure 6:**
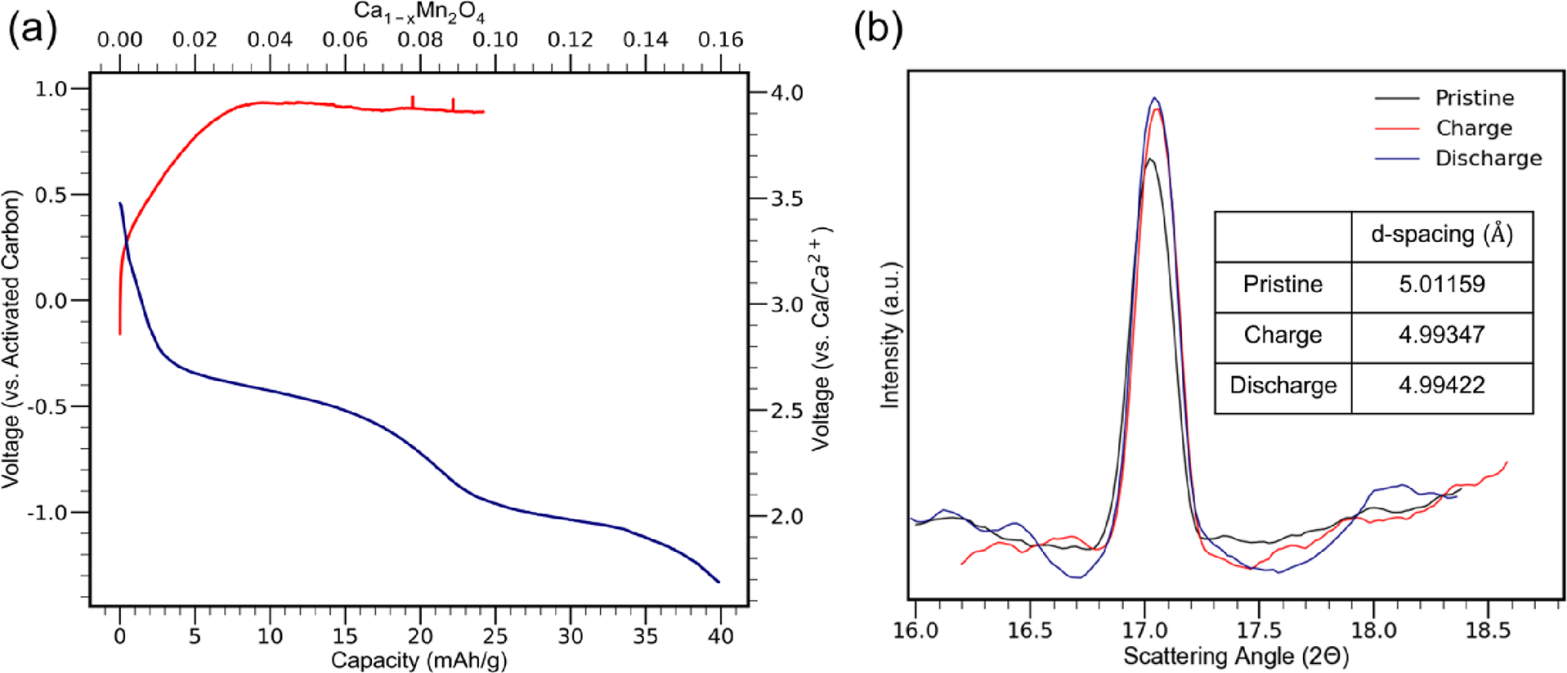
In situ cell cycling of CaMn_2_O_4_. (a) Voltage–capacity curves of CaMn_2_O_4_. (b) XRD of CaMn_2_O_4_ during charge and discharge within the in situ cell (inset of interplanar spacing during charge and discharge).

In situ XRD measurements made of CaMn_2_O_4_ were also performed on CaMn_2_O_4_. Freestanding electrodes of CaMn_2_O_4_ and activated carbon were assembled into the in situ XRD cell. Charge and discharge studies were performed on CaMn_2_O_4_ at a C-rate of C/200 (Figure 6a). The charging of CaMn_2_O_4_ achieved approximately 25 mAh/g of capacity, which would be 10% of calcium within the cathode. Discharge of CaMn_2_O_4_ exhibits a two-tiered reinsertion of calcium into the cathode, consistent with phase transformations that would be expected in the host structure.^44,59,60^ Based on the magnesium literature, the first plateau of the discharge curve would be the reduction of manganese from the +4 to +3 oxidation states. The second plateau would be attributed to the reduction of manganese from +3 to +2 oxidation states along with the activated carbon counter electrode forming a double layer capacitance.^46,48,49^ Following the charge and discharge of CaMn_2_O_4_, the XRD of the cell was measured. Structural changes of the (020) plane in CaMn_2_O_4_ are the most notable development with the in situ XRD pattern (Figure 6b). A shift of the (020) peak from 17.02° to 17.04° is consistent with a decrease in the *d*-spacing of CaMn_2_O_4_ from 5.01159 to 4.99347 Å as it is charged. Factors such as sample displacement were accounted for in the XRD measurements by having an adjustable stand with the in situ cell and maintaining the same sample height for each measurement. Additionally, there is an increase in the (020) intensity, consistent with previous findings on a decalciated structure.^40^ These findings are also consistent with DFT calculations performed on CaMn_2_O_4_. DFT calculations project a shift of the (020) lattice plane from 17.45° to 17.64°. Differences of the 2θ angles between experimental and theoretical predictions would be influenced by the glassy carbon window of the in situ cell. The DFT predictions for the changes in the *d*-spacing are a bit more substantial with changes from 5.07857 to 5.0235 Å as the electrode is charged. While there is a slight shift in the CaMn_2_O_4_ (020) reflection toward a lower angle, indicative of an increase in the interplanar spacing, there is no decrease in the intensity of the (020) signal, outlining a limited effect on reinserting calcium back into the CaMn_2_O_4_ cathode. A large portion of the capacity reported from the reinsertion is likely due to activated carbon forming a double layer capacitance.^40,48,49^

## Conclusions

Our study provides important insights into the intercalation chemistry of calcium-ion batteries using postspinel CaMn_2_O_4_ as a cathode material, which can offer an alternative to lithium-ion batteries in the future. We have shown the successful synthesis and characterization of postspinel CaMn_2_O_4_. Cyclic voltammetry measurements outlined the redox activity of the CaMn_2_O_4_ electrodes. In situ EIS measurements revealed the rapid increase in charge transfer resistance that builds within the electrode and leads to limited capacities during cycling. A maximum charge of CaMn_2_O_4_ at a rate of C/200 yielded a capacity of 100 mAh/g that could be extracted from the electrode. Structural changes of CaMn_2_O_4_ post charging with XRD revealed several features that aligned with DFT calculations made of decalciated CaMn_2_O_4_. Additional techniques involving XPS confirmed an increase in the average oxidation state of manganese, further supporting the redox activity of the CaMn_2_O_4_ electrode. Coin cell cycling demonstrated the capability of CaMn_2_O_4_ to reversibly cycle calcium at higher C/33 rates. The electrochemical activity of the coin cell, along with its capacity to function as a high-voltage cathode when measured against Ca/Ca^2+^, yielded an energy density of 205 Wh/kg. Results from in situ cell testing revealed a shift of the (020) reflection toward higher diffraction angles, outlining a decrease in the interplanar spacing that would occur from the removal of calcium and is consistent with theoretical calculations on CaMn_2_O_4_. Further evidence of the calcium extraction with the in situ experiment is the increase in the (020) reflection intensity, which was also projected with DFT calculations. Future work with in situ cell testing would have to include strategies to increase the active material with the working electrode. The intensities of several CaMn_2_O_4_ reflections were minimal due to the amount of active material within the electrode. Maximizing the amount of active material in the working electrode will not only increase the charge capacities but also improve the signal intensity of CaMn_2_O_4_, allowing for more detailed analysis of the structural changes to the electrode as it is cycled within the in situ cell.

The successful demonstration of the CaMn_2_O_4_ postspinel as a viable cathode material for calcium-ion batteries represents a significant breakthrough in the search for alternatives to lithium-ion batteries. Our findings indicate that the CaMn_2_O_4_ postspinel exhibits intercalation capacities and redox activity, with structural developments supporting its effectiveness as an electrode material. While the diffusion kinetics of calcium remain a limiting factor for achieving larger capacities, our study was able to accomplish all experiments at room temperature, providing a strong foundation for future optimization. These results establish a new baseline for the functionality of the CaMn_2_O_4_ postspinel and pave the way for further exploration of its potential as a high-performance cathode material. Further studies could investigate the use of other transition metals in the CaMn_2_O_4_ host to optimize the crystal structure and facilitate the more substantial diffusion of calcium.

## Supporting Information Available

Cyclic voltammetry of the Ag/Ag^+^ reference electrode, OCV of activated carbon, linear stability window of electrolyte, schematic of the in situ cell, in situ cell inside Rigaku Miniflex XRD, Nyquist plot of in situ EIS data, voltage profile of CaMn_2_O_4_ and convex hull from DFT calculations, structural models of deinserted Ca_1–*x*_Mn_2_O_4_ with *x* = 0.0, 0.25, 0.5, 0.75, and 1.0, theoretical XRD profiles of Ca_1–*x*_Mn_2_O_4_ deinsertion with *x* = 0.0, 0.25, 0.5, 0.75, and 1.0, atomic *x*, *y*, and *z* coordinates and occupancy of Ca_1–*x*_Mn_2_O_4_ deinsertion with *x* = 0.0, 0.25, 0.5, 0.75, and 1.0, Rietveld refinement of CaMn_2_O_4_ post maximum charge and discharge, refined parameters for CaMn_2_O_4_, Mn_3_O_4_, and MnO, experimental XRD of CaMn_2_O_4_ from maximum charge, theoretical XRD of CaMn_2_O_4_ from 25% decalciation, galvanostatic cycling of CaMn_2_O_4_ at C/39, and XRD of CaMn_2_O_4_ post coin cell cycling (PDF)
