# Supplementary material for: Exploring Calcium Manganese Oxide as a Promising Cathode Material for Calcium-Ion Batteries
Source: Chem Mater. 2023 Oct 6;35(20):8371–81. doi: 10.1021/acs.chemmater.3c00659 (PMC10601472; doi:10.1021/acs.chemmater.3c00659)
Supplement: Supplementary file 1 — cm3c00659_si_001.pdf [file cm3c00659_si_001.pdf]

SUPPLEMENTAL INFORMATION

Exploring Calcium Manganese Oxide as a Promising Cathode Material for Calcium-Ion Batteries

Paul Alexis Chando,[1] Sihe Chen[2], Jacob Matthew Shellhamer, [1] Elizabeth Wall, [1] Xinlu Wang,[1] Robson Schuarca,[1] Manuel Smeu[2], Ian Dean Hosein[1*]

1. Syracuse University, Department of Biomedical and Chemical Engineering, Syracuse, NY, 13244
2. Binghamton University State University of New York, Department of Physics, Binghamton, NY, 13902

*Corresponding author: idhosein@syr.edu

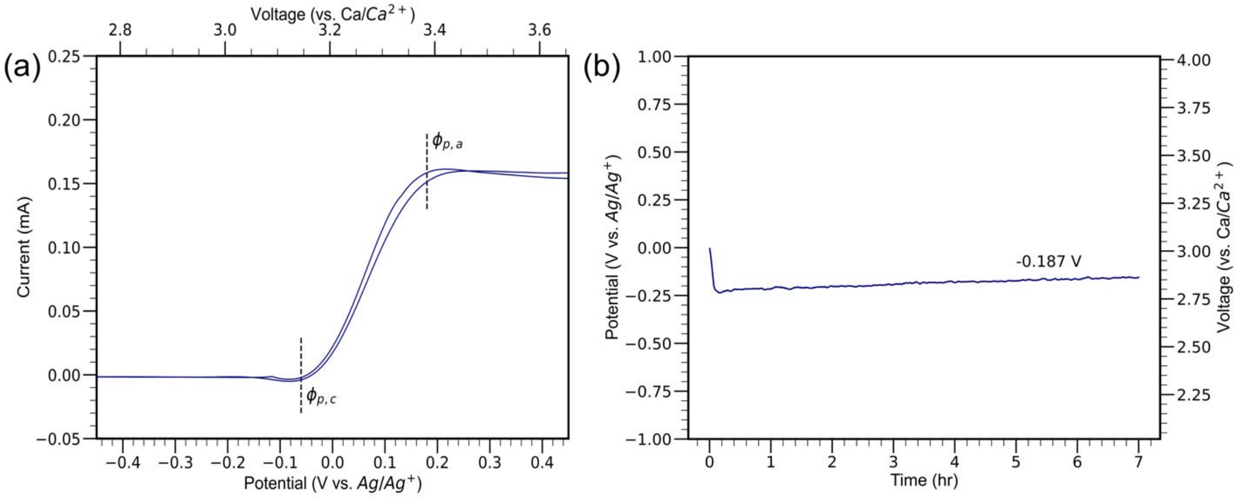

Figure S1 - Calibration of reference electrodes a) CV test of Ag/Ag^+ reference electrode (0.01 M $AgNO_3$ in 0.5 M $Ca(TFSI)_2$ DME) with 50 mM ferrocene dissolved in 0.5 M $Ca(TFSI)_2$ in DME with Pt working electrode and AC counter electrode b) OCV of the activated carbon is -0.187 V vs. Ag/Ag^+

Figure S1a shows the cyclic voltammetry (CV) of a three-electrode system with 50 mM ferrocene dissolved in $Ca(TFSI)_2$ in DME. The working electrode was platinum while the counter and reference electrodes were activated carbon and Ag/Ag^+, respectively. The ferrocene potential was calculated using equation (1)

$$\phi_{Fc/Fc^+} = \frac{\phi_{p,c} + \phi_{p,a}}{2} \qquad (1)$$

with ϕ_{Fc/Fc^+} being the measured potential of the ferrocene/ferrocenium reaction. $\phi_{p,c}$ and $\phi_{p,a}$ were the cathodic and anodic potentials, respectively. The results from the CV yielded a redox potential at 0.065 V. The value of the ferrocene reaction against the standard hydrogen electrode is 0.4 V. Using the results of the ferrocene CV and the established 0.4 V (vs. SHE) redox reaction, the potential of the Ag/Ag^+ reference electrode was adjusted from its standard reduction potential of 0.8 V to 1.135 V. The calibration also establishes that the Ca/Ca^{2+} redox reaction would occur at -3.205 V vs Ag/Ag^+. Following the calibration of the Ag/Ag^+ reference electrode, an open circuit voltage (OCV) measurement of the activated carbon was performed and found the potential to be -0.187 V (vs Ag/Ag+). (Figure S1b) Using the OCV, the voltage of the activated carbon would be 3.018 V (vs Ca/Ca^{2+}). Values from the ferrocene CV and activated carbon OCV are in close agreement with previous studies on reference electrode calibrations.[1,2]

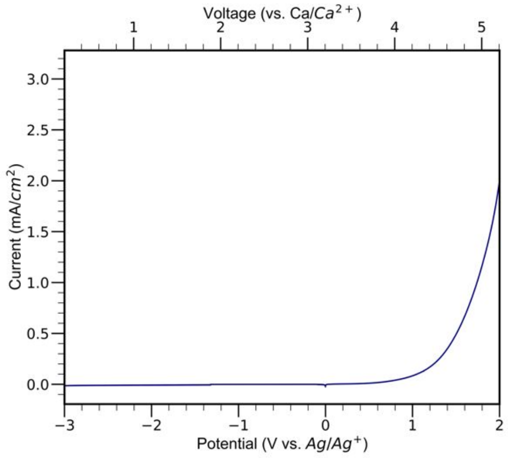

Figure S2 - Linear Stability Window of $Ca(TFSI)_2$ in DME using 316 stainless steel blocking and calcium nonblocking electrode at 0.5 mV/s

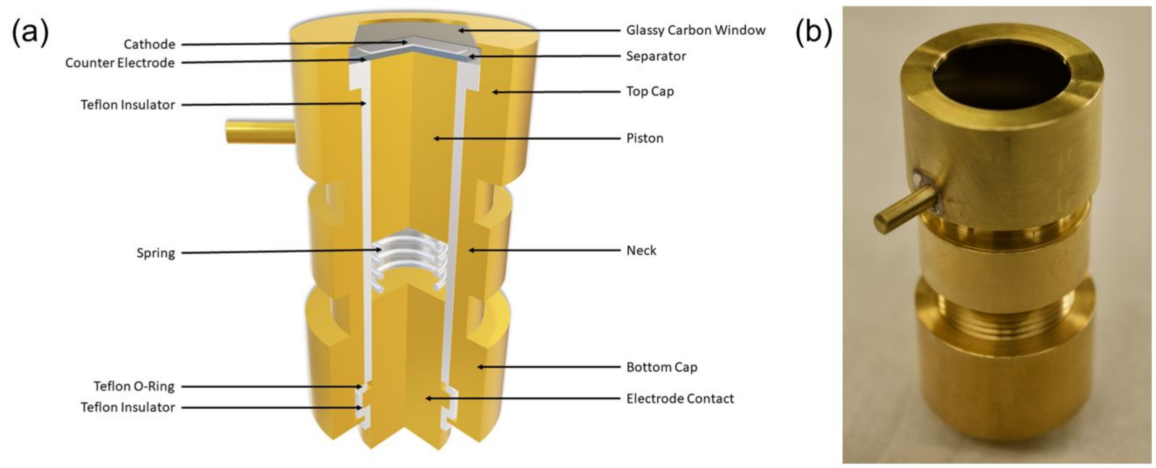

Figure S3 - In-Situ cell a) Schematic of in-situ cell used for $CaMn_2O_4$ b) photo of the in-situ cell assembled

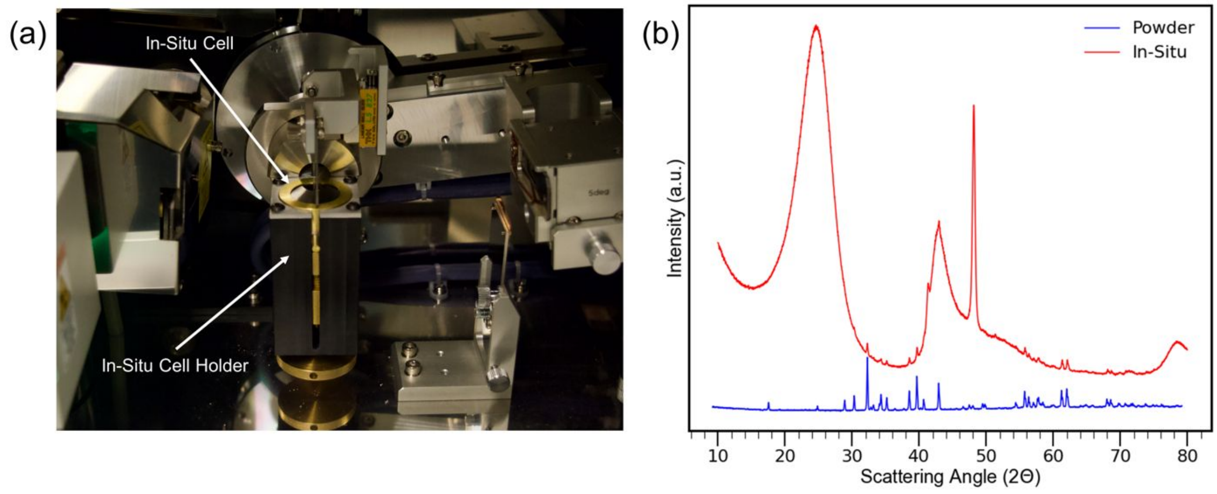

Figure S4 - a) Installation of in-situ cell inside of the Rigaku XRD b) baseline XRD scan of $CaMn_2O_4$ inside of in-situ cell.

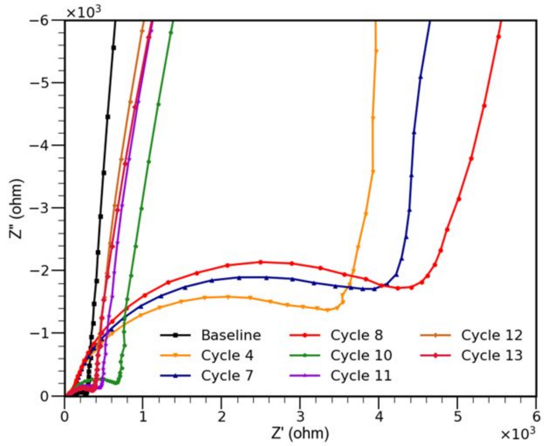

Figure S5 - Nyquist plots of impedance for $CaMn_2O_4$ after charge and discharge cycling

After the DFT calculations were completed, structure files were generated using VASP. These files were opened with VESTA[3] and XRD patterns were generated using powder diffraction pattern functionality. The simulation incident light has wavelength of 1.54059 Å (X-ray) and the XRD plot was generated based on atomic positions.

The method for simulating the XRD diffraction pattern required the use of eq. 2. After performing Fourier transformation of the structural information, the phase of incident light and the structure were combined. The intensity of reflected light can be calculated using structure factor,

$$I(q) = f^2 \sum_{i=1}^{N} e^{(-iq \cdot R_i)}, \qquad (2)$$

where R_i is the atomic position, f is the atomic form factor, and q is the scattering vector. A plot of intensity with respect to angle 2θ was generated. The results for the theoretical XRD patterns from $CaMn_2O_4$ at 0% and 25% decalciation are outlined in Figure S10b. The generated lattice parameters, coordinates and theoretical XRD patterns from DFT calculations are summarized in Figures S7 & S8 and Tables S1-S8. Structural information in Tables S2-S8 used P1 cell symmetry.

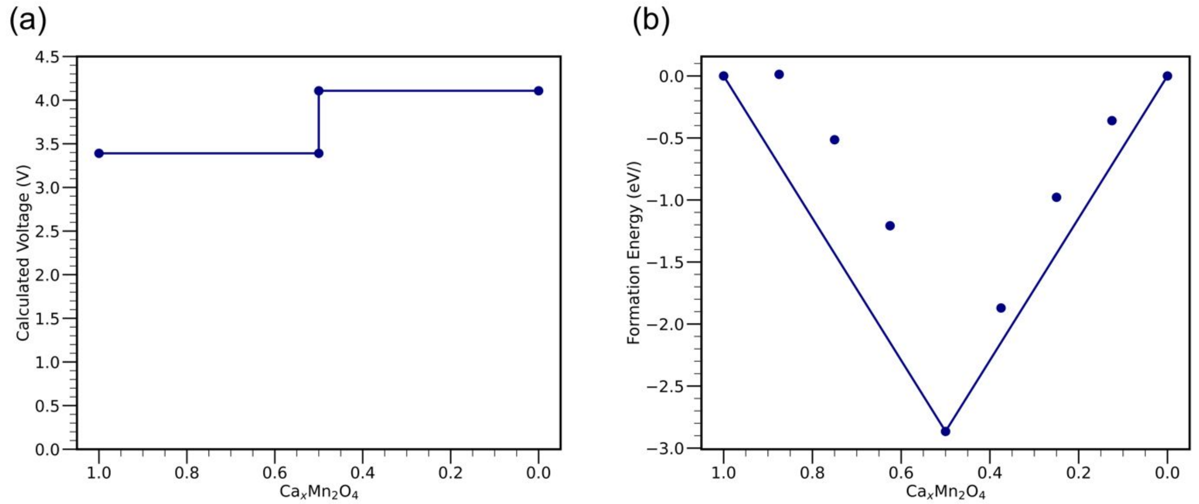

Figure S6 - DFT Calculations of $CaMn_2O_4$ a) Voltage profile of $CaMn_2O_4$ as calcium is removed. Voltage values were calculated with stable concentrations of calcium in $CaMn_2O_4$ with the convex hull. b) Convex hull of $CaMn_2O_4$. Three concentrations of calcium were on the hull and connected by the line.

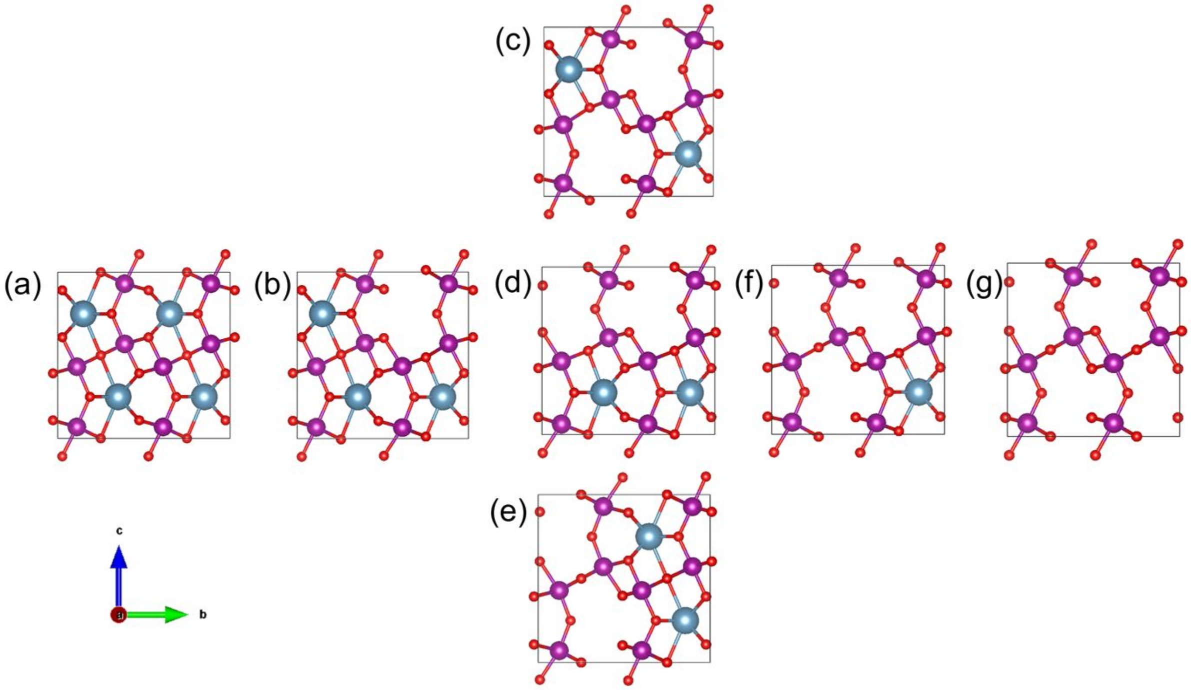

Figure S7 - Structural models of $Ca_{1-x}Mn_2O_4$ deinsertion a) x = 0 b) x = 0.25 c) x = 0.5 d) x = 0.5 e) x = 0.5 f) x = 0.75 g) x = 1.0

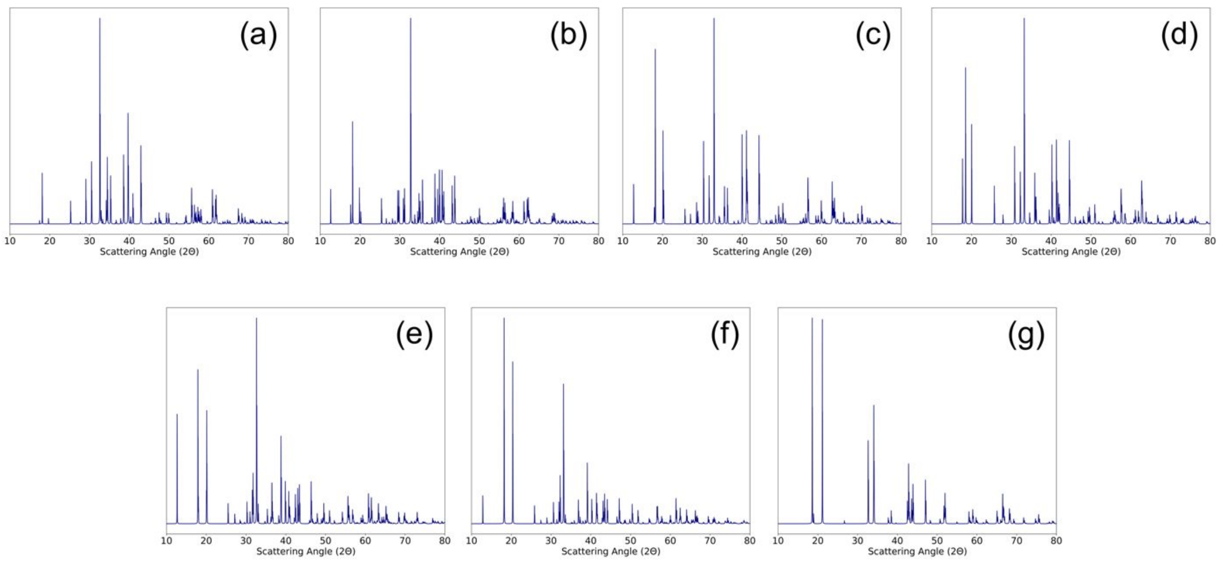

Figure S8 - Theoretical XRD patterns of $Ca_{1-x}Mn_2O_4$ deinsertion a) x = 0 b) x = 0.25 c) x = 0.5 d) x = 0.5 e) x = 0.5 f) x = 0.75 g) x = 1.0

Table S1 - Calculated lattice parameters of $Ca_{1-x}Mn_2O_4$ deinsertion a) x = 0 b) x = 0.25 c) x = 0.5 d) x = 0.5 e) x = 0.5 f) x = 0.75 g) x = 1.0

	**(a)**	**(b)**	**(c)**	**(d)**	**(e)**	**(f)**	**(g)**
**Space Group**	Pbcm	Pm	$P2_1/m$	$Pmc2_1$	Pma2	Pm	Cmcm
**a (Å)**	3.21696	3.15752	3.02402	3.02795	3.08506	2.98366	2.86492
**b (Å)**	10.15714	10.04844	9.87361	9.99746	9.88520	9.78194	9.35579
**c (Å)**	9.78279	9.77412	9.90640	9.58717	9.75041	9.74097	9.53471
**alpha**	90	90	90	90	90	90	90
**beta**	90	90	90	90	90	90	90
**gamma**	90	90.9703	94.9005	90	90	95.3300	90
**Volume ($Å^3$)**	319.6542	310.0698	294.7035	290.2206	297.3530	283.0702	255.5645

Table S2 - Atomic x, y, z coordinates and occupancy of $Ca_{1-x}Mn_2O_4$ deinsertion with x = 0 (a)

Element	x	y	z	Occupancy	Biso	Symmetry
Mn1	0.20741	0.38861	0.56872	1	0	1a
Mn2	0.79259	0.61139	0.43128	1	0	1a
Mn3	0.79259	0.61139	0.06872	1	0	1a
Mn4	0.20741	0.38861	0.93128	1	0	1a
Mn5	0.79259	0.88861	0.93128	1	0	1a
Mn6	0.20741	0.11139	0.06872	1	0	1a
Mn7	0.20741	0.11139	0.43128	1	0	1a
Mn8	0.79259	0.88861	0.56872	1	0	1a
Ca1	0.31975	0.64956	0.75000	1	0	1a
Ca2	0.68025	0.35044	0.25000	1	0	1a
Ca3	0.68025	0.14956	0.75000	1	0	1a
Ca4	0.31975	0.85044	0.25000	1	0	1a
O1	0.81210	0.81293	0.75000	1	0	1a
O2	0.18790	0.18707	0.25000	1	0	1a
O3	0.18790	0.31293	0.75000	1	0	1a
O4	0.81210	0.68707	0.25000	1	0	1a
O5	0.79989	0.52693	0.60840	1	0	1a
O6	0.20011	0.47307	0.39160	1	0	1a
O7	0.20011	0.47307	0.10840	1	0	1a
O8	0.79989	0.52693	0.89160	1	0	1a
O9	0.20011	0.02693	0.89160	1	0	1a
O10	0.79989	0.97307	0.10840	1	0	1a

O11	0.79989	0.97307	0.39160	1	0	1a
O12	0.20011	0.02693	0.60840	1	0	1a
O13	0.41369	0.75000	0.50000	1	0	1a
O14	0.58631	0.25000	0.50000	1	0	1a
O15	0.58631	0.25000	0.00000	1	0	1a
O16	0.41369	0.75000	0.00000	1	0	1a

Table S3 - Atomic x, y, z coordinates and occupancy of $Ca_{1-x}Mn_2O_4$ deinsertion with x = 0.25 (b)

Element	x	y	z	Occupancy	Biso	Symmetry
Mn1	0.22867	0.39685	0.57301	1	0	1a
Mn2	0.78981	0.61431	0.43390	1	0	1a
Mn3	0.78981	0.61431	0.06610	1	0	1a
Mn4	0.22867	0.39685	0.92699	1	0	1a
Mn5	0.78378	0.88411	0.92890	1	0	1a
Mn6	0.22606	0.11148	0.06818	1	0	1a
Mn7	0.22606	0.11148	0.43182	1	0	1a
Mn8	0.78378	0.88411	0.57110	1	0	1a
Ca1	0.69770	0.35446	0.25000	1	0	1a
Ca2	0.69131	0.14775	0.75000	1	0	1a
Ca3	0.32500	0.84975	0.25000	1	0	1a
O1	0.77912	0.81724	0.75000	1	0	1a
O2	0.20186	0.18852	0.25000	1	0	1a
O3	0.20109	0.31590	0.75000	1	0	1a
O4	0.81657	0.68402	0.25000	1	0	1a

O5	0.73681	0.50649	0.60359	1	0	1a
O6	0.20313	0.47443	0.39430	1	0	1a
O7	0.20313	0.47443	0.10571	1	0	1a
O8	0.73681	0.50649	0.89641	1	0	1a
O9	0.20586	0.02673	0.89109	1	0	1a
O10	0.80158	0.97480	0.10688	1	0	1a
O11	0.80158	0.97480	0.39312	1	0	1a
O12	0.20586	0.02673	0.60891	1	0	1a
O13	0.39520	0.75136	0.48795	1	0	1a
O14	0.61399	0.25621	0.50476	1	0	1a
O15	0.61399	0.25621	0.99524	1	0	1a
O16	0.39520	0.75136	0.01205	1	0	1a

Table S4 - Atomic x, y, z coordinates and occupancy of $Ca_{1-x}Mn_2O_4$ deinsertion with x = 0.5 (c)

Element	x	y	z	Occupancy	Biso	Symmetry
Mn1	0.22519	0.39463	0.57167	1	0	1a
Mn2	0.77481	0.60537	0.42833	1	0	1a
Mn3	0.77481	0.60537	0.07167	1	0	1a
Mn4	0.22519	0.39463	0.92833	1	0	1a
Mn5	0.73099	0.88570	0.92318	1	0	1a
Mn6	0.26901	0.11430	0.07682	1	0	1a
Mn7	0.26901	0.11430	0.42318	1	0	1a
Mn8	0.73099	0.88570	0.57682	1	0	1a
Ca1	0.69825	0.14808	0.75000	1	0	1a

Ca2	0.30175	0.85192	0.25000	1	0	1a
O1	0.65418	0.82085	0.75000	1	0	1a
O2	0.34582	0.17915	0.25000	1	0	1a
O3	0.22284	0.32377	0.75000	1	0	1a
O4	0.77716	0.67623	0.25000	1	0	1a
O5	0.75370	0.51567	0.60221	1	0	1a
O6	0.24630	0.48433	0.39779	1	0	1a
O7	0.24630	0.48433	0.10221	1	0	1a
O8	0.75370	0.51567	0.89779	1	0	1a
O9	0.20665	0.02964	0.89409	1	0	1a
O10	0.79335	0.97036	0.10591	1	0	1a
O11	0.79335	0.97036	0.39409	1	0	1a
O12	0.20665	0.02964	0.60591	1	0	1a
O13	0.32231	0.73033	0.47593	1	0	1a
O14	0.67769	0.26967	0.52407	1	0	1a
O15	0.67769	0.26967	0.97593	1	0	1a
O16	0.32231	0.73033	0.02407	1	0	1a

Table S5 - Atomic x, y, z coordinates and occupancy of $Ca_{1-x}Mn_2O_4$ deinsertion with x = 0.5 (d)

Element	x	y	z	Occupancy	Biso	Symmetry
Mn1	0.18099	0.38648	0.57847	1	0	1a
Mn2	0.72385	0.61251	0.43775	1	0	1a
Mn3	0.72385	0.61251	0.06225	1	0	1a
Mn4	0.18099	0.38648	0.92153	1	0	1a

Mn5	0.81901	0.88648	0.92153	1	0	1a
Mn6	0.27615	0.11252	0.06225	1	0	1a
Mn7	0.27615	0.11252	0.43775	1	0	1a
Mn8	0.81901	0.88648	0.57847	1	0	1a
Ca1	0.69049	0.35705	0.25000	1	0	1a
Ca2	0.30951	0.85705	0.25000	1	0	1a
O1	0.82356	0.80820	0.75000	1	0	1a
O2	0.21395	0.18646	0.25000	1	0	1a
O3	0.17644	0.30820	0.75000	1	0	1a
O4	0.78605	0.68646	0.25000	1	0	1a
O5	0.67964	0.50442	0.61043	1	0	1a
O6	0.16910	0.47928	0.39630	1	0	1a
O7	0.16910	0.47928	0.10370	1	0	1a
O8	0.67964	0.50442	0.88957	1	0	1a
O9	0.32036	0.00442	0.88957	1	0	1a
O10	0.83090	0.97928	0.10370	1	0	1a
O11	0.83090	0.97928	0.39630	1	0	1a
O12	0.32036	0.00442	0.61043	1	0	1a
O13	0.34901	0.76704	0.49946	1	0	1a
O14	0.65099	0.26704	0.49946	1	0	1a
O15	0.65099	0.26704	0.00054	1	0	1a
O16	0.34901	0.76704	0.00054	1	0	1a

Table S6 - Atomic x, y, z coordinates and occupancy of $Ca_{1-x}Mn_2O_4$ deinsertion with x = 0.5 (e)

Element	x	y	z	Occupancy	Biso	Symmetry
Mn1	0.21500	0.37853	0.57067	1	0	1a
Mn2	0.76363	0.60297	0.42849	1	0	1a
Mn3	0.76363	0.60297	0.07152	1	0	1a
Mn4	0.21500	0.37853	0.92933	1	0	1a
Mn5	0.76363	0.89703	0.92848	1	0	1a
Mn6	0.21500	0.12147	0.07067	1	0	1a
Mn7	0.21500	0.12147	0.42933	1	0	1a
Mn8	0.76363	0.89703	0.57152	1	0	1a
Ca1	0.29500	0.64494	0.75000	1	0	1a
Ca2	0.29500	0.85506	0.25000	1	0	1a
O1	0.78649	0.81599	0.75000	1	0	1a
O2	0.20512	0.18771	0.25000	1	0	1a
O3	0.20512	0.31229	0.75000	1	0	1a
O4	0.78649	0.68401	0.25000	1	0	1a
O5	0.79038	0.52429	0.60737	1	0	1a
O6	0.25860	0.49084	0.39764	1	0	1a
O7	0.25860	0.49084	0.10236	1	0	1a
O8	0.79038	0.52429	0.89263	1	0	1a
O9	0.25860	0.00916	0.89764	1	0	1a
O10	0.79038	0.97571	0.10737	1	0	1a
O11	0.79038	0.97571	0.39263	1	0	1a
O12	0.25860	0.00916	0.60236	1	0	1a

O13	0.34353	0.75000	0.50000	1	0	1a
O14	0.63219	0.25000	0.50000	1	0	1a
O15	0.63219	0.25000	0.00000	1	0	1a
O16	0.34353	0.75000	0.00000	1	0	1a

Table S7 - Atomic x, y, z coordinates and occupancy of $Ca_{1-x}Mn_2O_4$ deinsertion with x = 0.75 (f)

**Element**	**x**	**y**	**z**	**Occupancy**	**Biso**	**Symmetry**
Mn1	0.20158	0.38870	0.57660	1	0	1a
Mn2	0.76135	0.60601	0.43231	1	0	1a
Mn3	0.76135	0.60601	0.06769	1	0	1a
Mn4	0.20158	0.38870	0.92340	1	0	1a
Mn5	0.74277	0.89470	0.91827	1	0	1a
Mn6	0.29525	0.11841	0.07202	1	0	1a
Mn7	0.29525	0.11841	0.42798	1	0	1a
Mn8	0.74277	0.89470	0.58173	1	0	1a
Ca1	0.30680	0.85120	0.25000	1	0	1a
O1	0.72435	0.81364	0.75000	1	0	1a
O2	0.29349	0.18962	0.25000	1	0	1a
O3	0.17474	0.31232	0.75000	1	0	1a
O4	0.77044	0.67759	0.25000	1	0	1a
O5	0.73506	0.51271	0.60860	1	0	1a
O6	0.22913	0.48584	0.39946	1	0	1a
O7	0.22913	0.48584	0.10054	1	0	1a
O8	0.73506	0.51271	0.89140	1	0	1a

O9	0.27415	0.01325	0.89353	1	0	1a
O10	0.81030	0.97619	0.10443	1	0	1a
O11	0.81030	0.97619	0.39557	1	0	1a
O12	0.27415	0.01325	0.60647	1	0	1a
O13	0.31212	0.73571	0.48077	1	0	1a
O14	0.66214	0.27189	0.51390	1	0	1a
O15	0.66214	0.27189	0.98610	1	0	1a
O16	0.31212	0.73571	0.01923	1	0	1a

Table S8 - Atomic x, y, z coordinates and occupancy of $Ca_{1-x}Mn_2O_4$ deinsertion with x = 1.0 (g)

Element	x	y	z	Occupancy	Biso	Symmetry
Mn1	0.24956	0.38384	0.57700	1	0	1a
Mn2	0.75044	0.61616	0.42300	1	0	1a
Mn3	0.75044	0.61616	0.07700	1	0	1a
Mn4	0.24956	0.38384	0.92300	1	0	1a
Mn5	0.75044	0.88384	0.92300	1	0	1a
Mn6	0.24956	0.11616	0.07700	1	0	1a
Mn7	0.24956	0.11616	0.42300	1	0	1a
Mn8	0.75044	0.88384	0.57700	1	0	1a
O1	0.75182	0.80217	0.75000	1	0	1a
O2	0.24818	0.19782	0.25000	1	0	1a
O3	0.24818	0.30217	0.75000	1	0	1a
O4	0.75182	0.69783	0.25000	1	0	1a
O5	0.74965	0.51078	0.60865	1	0	1a

O6	0.25035	0.48922	0.39135	1	0	1a
O7	0.25035	0.48922	0.10865	1	0	1a
O8	0.74965	0.51078	0.89135	1	0	1a
O9	0.25035	0.01078	0.89135	1	0	1a
O10	0.74965	0.98922	0.10865	1	0	1a
O11	0.74965	0.98922	0.39135	1	0	1a
O12	0.25035	0.01078	0.60865	1	0	1a
O13	0.25035	0.75000	0.50000	1	0	1a
O14	0.74965	0.25000	0.50000	1	0	1a
O15	0.74965	0.25000	0.00000	1	0	1a
O16	0.25035	0.75000	0.00000	1	0	1a

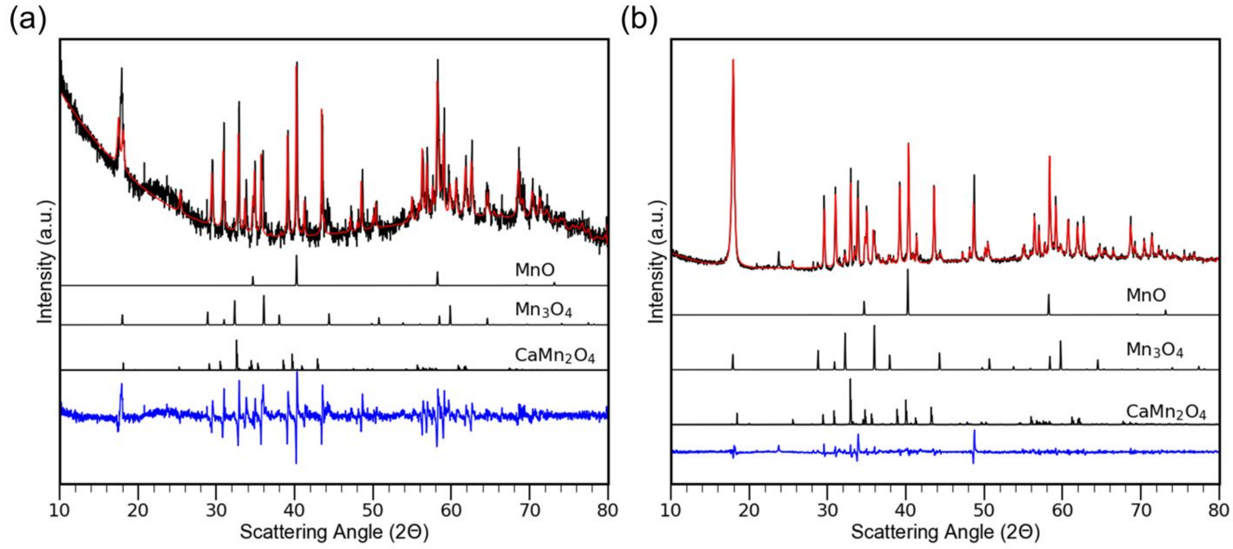

Figure S9 - Rietveld refinement of $CaMn_2O_4$ cathode a) post-maximum charge b) post-discharge

Table S9 - Refined parameters for $CaMn_2O_4$

	a (Å)	Deviation (Å)	b (Å)	Deviation (Å)	c (Å)	Deviation (Å)
**Pristine**	3.156676	0.000119	9.988452	0.000457	9.675391	0.000396
**Charged**	3.154765	0.000302	9.978689	0.001508	9.666233	0.00109
**Discharged**	3.158088	0.000311	9.997047	0.001092	9.678633	0.000979

Table S10 - Refined parameters for Mn_3O_4

	a (Å)	Deviation (Å)	b (Å)	Deviation (Å)	c (Å)	Deviation (Å)
**Charged**	5.761503	0.000405	5.761503	0	9.464502	0.002912
**Discharged**	5.753304	0.001810	5.753304	0	9.481883	0.003148

Table S11 - Refined parameters for MnO

	a (Å)	Deviation (Å)	b (Å)	Deviation (Å)	c (Å)	Deviation (Å)
**Charged**	4.48863	0.001023	4.48863	0	4.48863	0
**Discharged**	4.46448	0.001569	4.46448	0	4.46448	0

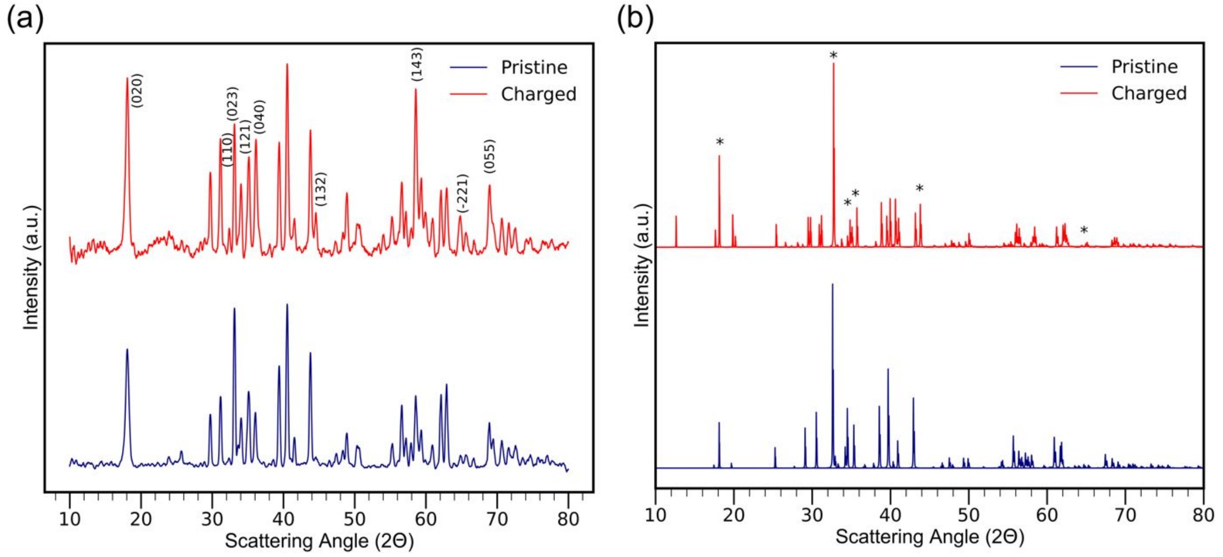

Figure S10 - XRD changes with $CaMn_2O_4$ from maximum charge a) Full experimental XRD of $CaMn_2O_4$ from its pristine electrode and after maximum charge of the $CaMn_2O_4$ b) Theoretical XRD of the $CaMn_2O_4$ with 25% vacancies from the pristine electrode

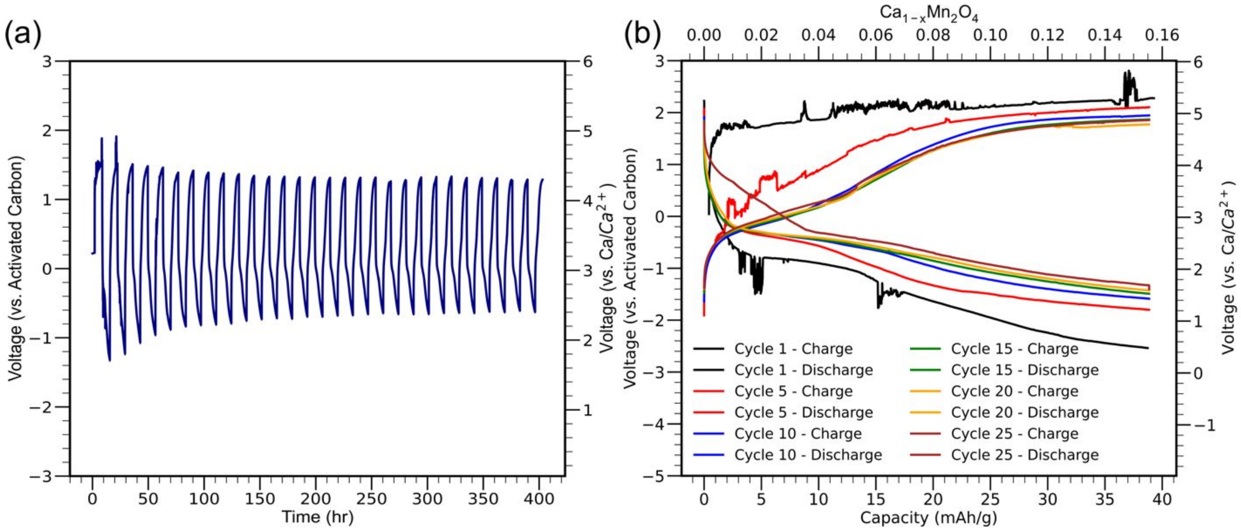

Figure S11 - Galvanostatic cycling of the $CaMn_2O_4$ at C/39 a) Voltage-time curve of 30 cycles of testing b) Capacity-voltage curves of selected cycles of $CaMn_2O_4$

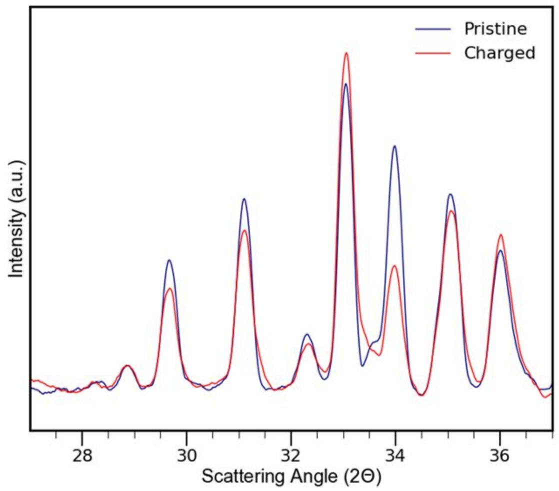

Figure S12 - XRD of the $CaMn_2O_4$ before after galvanostatic cycling of the $CaMn_2O_4$

Supplemental References

1. Zuo, C.; Xiong, F.; Wang, J.; An, Y.; Zhang, L.; An, Q. MnO $_2$ Polymorphs as Cathode Materials for Rechargeable Ca-ion Batteries. *Adv. Funct. Mater.* **2022**, 2202975.
2. Xu, Z.-L.; Park, J.; Wang, J.; Moon, H.; Yoon, G.; Lim, J.; Ko, Y.-J.; Cho, S.-P.; Lee, S.-Y.; Kang, K. A New High-Voltage Calcium Intercalation Host for Ultra-Stable and High-Power Calcium Rechargeable Batteries. *Nat. Commun.* **2021**, *12* (1), 3369.
3. Momma, K. and Izumi, F. (2008) VESTA: A Three-Dimensional Visualization System for Electronic and Structural Analysis. Journal of Applied Crystallography, 41, 653-658. http://dx.doi.org/10.1107/S0021889808012016
